# The nosebleed fever outbreak in Iraq: Challenges, efforts and recommendations

**DOI:** 10.1016/j.amsu.2022.104077

**Published:** 2022-06-25

**Authors:** Shkaib Ahmad, Narmeen Hashmi, Javeria Arif Siddiqui, Amna Siddiqui, Shoaib Ahmad, Hashim Talib Hashim, Mohammad Yasir Essar

**Affiliations:** DHQ Teaching Hospital Dg Khan, Pakistan; Faculty of Medicine, Sindh Medical College, Jinnah Sindh Medical University, Karachi, Pakistan; Department of MBBS, Karachi Medical and Dental College, Karachi, 74700, Pakistan; District Headquarter Teaching Hospital, Faisalabad, Pakistan; University of Baghdad, College of Medicine, Iraq; Kabul University of Medical Sciences, Kabul, Afghanistan

Crimean-Congo hemorrhagic fever virus (CCHFV) is a virulent disease without any approved therapeutics or vaccination and treatment being supportive mainly. It was first recognized in 1944 in Crimean Peninsula when an outbreak occurred within military personnel, thus the condition named Crimean hemorrhagic fever. Meanwhile, Dr. Courtois from Congo isolated a virus from a febrile teenage boy using a newborn mouse in 1956 and named the virus Congo virus strain V3011, combining the names to CCHFV [1,2].

Various lab tests are used to diagnose CCHF including antigen-capture enzyme-linked immunosorbent assay (ELISA), real time polymerase chain reaction (RT-PCR), virus isolation attempts, and detection of antibody by ELISA (IgG and IgM). During the acute phase of the disease, the combination of viral antigen detection, viral RNA sequence in blood and virus isolation from blood, along with a clinical history can aid the diagnosis. In the later cases antibodies can be found but antigen, viral RNA and virus are no more detectable [2,3].

Iraq, an eastern Mediterranean country, where CCHF is endemic reported its first case in 1979 when the disease was first diagnosed in ten patients. In the 1989–2009 duration six cases were reported; 11 cases in 2010; in 2018 three fatal cases were reported in 2018. In 2021, 33 cases were confirmed including 13 deaths. In the recent outbreak, 212 cases have been reported to the WHO from the Iraqi health authorities in the span of 5 months from January 2022 to May 2022, with most cases being reported in April a May. Out of 212 cases, 115 were suspected and 97 were confirmed using PCR by the Iraq Central Public Health Laboratory. Twenty-seven deaths occurred, of which 13 were laboratory confirmed cases, with a case fatality of 13% [4,5].

Further, Iraq has proportionately a significant population living in the village where subsistence farming is practised, in these settings, CCHF may be transmitted from domestic animals to humans. Sheep and cattle husbandry is very common in Iraq and studies have revealed that these animals are recurrently infested with tick species, mainly *Hylomma* species, the principal vector of CCHF resulting in its transmission irresistible. Secondly, the state is more prone to an increased incidence of CCHF cases due to the upcoming religious holiday, Eid al-Adha in July, because relatively more camels, cows, goats and sheep will be slaughtered during that period. Additionally, cross-border transmission is inevitable as a vast number of crowds will be migrating across the border because of official holidays. Thus, considering WHO's latest update of June 2022, on risk assessment for the state of Iraq underscores the vulnerability of the state to an endemic of CCHF [6,7].

The CCHFV RNA RT-PCR diagnostic test is most performed in reference laboratories as a consequence of which diagnostic reports may take 3 days or even longer become available, emphasizing the need for setting up an algorithm for the early identification and diagnosis of cases [7,8]. Moreover, there is yet no approved antiviral treatment for CCHF i.e. vaccines formulated so far [9]. In addition to existing aforementioned limitations in combating CCHF, the country already lags in diagnostic capacity, and outbreak response activities among its all governorates, that make the emergence of endemic foci of CCHF in the state of Iraq more predictable.

To control and alleviate the current outbreak of CCHF in Iraq, a variety of strategies must be implemented. Considering majority of the cases are seen in farmers, veterinarians, and people working with livestock, community awareness becomes an integral effort to prevent and control the spread of this disease [10,11].

Strengthening national and regional surveillance activities could allow identification and understanding of gaps and constraints faced in the control of this outbreak in specific geographical distributions [12]. Consequently, these disparities can be addressed and consolidated via programs specific to the needs identified. Furthermore, the onset of CCHF is quite sudden and by the time a diagnosis is reached, it is often too late. To combat this, the standardisation of case definitions may help to aid early diagnosis. The disparity exists due to little awareness of the early symptoms of CCHF leading to inappropriate management and therefore fluctuations in the mortality rate [12]. This mortality rate is also varied due to inconsistency in reporting cases. Some patients are reported after haemorrhage whilst others are reported before [12]. This variation highlights the gap in surveillance and the result of blurred case definitions. Although CCHF can be diagnosed by using real time polymerase chain reaction (RT-PCR) tests, results often take up to 3–5 days to become available [12]. An early diagnosis and supportive treatment via basic management of symptoms has proven to be integral for a better clinical outcome and is the keystone of outbreak control [12]. Therefore, until faster diagnostics tests for CCHF can be developed for primary care settings, standardisation of case definitions is essential in the effort for early diagnosis.

The most effective effort in the control of this outbreak is CCHF prevention. This can be achieved through various methods. Safe removal of ticks is an essential first step in the prevention of CCHF. Ticks should be removed straight up using tweezers and care should be taken not to twist or jerk the tick. This is followed by cleaning of the tick bite with soap and water or antiseptic. Furthermore, reducing human-to-human transmission is also intrinsic to CCHF prevention. Avoiding contact with diseased or deceased patients, as well as practicing good hand hygiene and seeking early treatment are all essential components to reducing transmission. Moreover, the infection must be controlled within the healthcare setting as well. Nosocomial outbreaks of CCHF were reported as a result of splashes and needle stick injuries, both of which are preventable routes [12,13]. This can be achieved by practicing extra caution and infection control measures to prevent contact with infected blood and bodily fluids, as well as contaminated surfaces. Finally, reducing ticks in farms and areas with livestock, through the use of pesticides like acaricide, and introducing quarantine measures for infected animals can help prevent and control the spread of CCHF.

## Ethical approval

None.

## Sources of funding

None.

## Author contribution

All authors contributed equally.

## Declaration of competing interest

None declared.

## Registration of research studies

1. Name of the registry:
2. Unique Identifying number or registration ID:
3. Hyperlink to your specific registration (must be publicly accessible and will be checked):

## Guarantor

N/A.

## Consent

N/A.
